# Accelerating Scientific Discovery Through Computation and Visualization III. Tight-Binding Wave Functions for Quantum Dots

**DOI:** 10.6028/jres.113.010

**Published:** 2008-06-01

**Authors:** James S. Sims, William L. George, Terence J. Griffin, John G. Hagedorn, Howard K. Hung, John T. Kelso, Marc Olano, Adele P. Peskin, Steven G. Satterfield, Judith Devaney Terrill, Garnett W. Bryant, Jose G. Diaz

**Affiliations:** Math and Computational Sciences Division (ITL), National Institute of Standards and Technology, Gaithersburg, MD 20899-8911; Atomic Physics Division (PL), National Institute of Standards and Technology, Gaithersburg, MD 20899-8423

**Keywords:** high-performance computing, MPI, nanotechnology, parallel computing, quantum dots, RAVE, tight-binding, virtual measurements, visualization

## Abstract

This is the third in a series of articles that describe, through examples, how the Scientific Applications and Visualization Group (SAVG) at NIST has utilized high performance parallel computing, visualization, and machine learning to accelerate scientific discovery. In this article we focus on the use of high performance computing and visualization for simulations of nanotechnology.

## 1. Introduction

This is the third in a series of articles [1, 2] that describe, through examples, how the Scientific Applications and Visualization Group (SAVG) at the National Institute of Standards and Technology (NIST) has utilized high performance parallel computing, visualization, and machine learning to accelerate scientific discovery. In this article we focus on the use of high performance computing and visualization for simulations of nanotechnology.

Research and development of nanotechnology, with applications ranging from smart materials to quantum computation to biolabs on a chip, has the highest national priority. Semiconductor nanoparticles, also known as nanocrystals and quantum dots, are one of the most intensely studied nanotechnology paradigms. Nanoparticles are typically 1 nm to 10 nm in size with a thousand to a million atoms. Precise control of particle size, shape and composition allows one to tailor charge distributions and control quantum effects to tailor properties completely different from the bulk and from small clusters. As a result of enhanced quantum confinement effects, nanoparticles act as artificial, man-made atoms with discrete electronic spectra that can be exploited as light sources for novel enhanced lasers, discrete components in nanoelectronics, qubits for quantum information processing and enhanced ultra-stable fluorescent labels for biosensors to detect, for example, cancers, malaria or other pathogens, and to do cell biology.

Semiconductor nanoparticles come in two kinds. Nanocrystals are small crystallites, 1 nm to 6 nm in dimension, typically made with colloidal chemistry, and containing up to 100 000 atoms [3]. Such nanoparticles are ideal for biosensor applications because they can be conjugated to be made soluble for in vivo and in vitro studies and can be functionalized to bind to specific biological structures [4]. At the same time, these nanocrystals can be used as the building blocks, linked together by coordinating ligands, to form nanoarchitectures for nanoelectronics, quantum computing and spintronics [5]. Larger semiconductor quantum dots can be formed by controlled epitaxial growth, typically using molecular beam epitaxy, to grow a low bandgap wetting layer and pyramidal or hemispherical dot, often InAs, embedded in a high bandgap material, usually GaAs. These dots are typically 10 nm to 20 nm wide and a few nm high with 100 000 to 1 000 000 atoms. These nanoparticles have compelling interest because they can be grown and integrated into current microelectronic devices for use in optoelectronics and electronics.

Initially, nanoparticles were modeled by continuum, effective mass theories, essentially treating the confined electrons and holes as “particles-in-a-box” with sophisticated models for the particle mass. Such approaches are easy to implement and work well enough for large systems where the atomic-scale details do not matter. More recently, atomic-scale theories, such as tight-binding theory and ab initio theory, have been used to model nanoparticles. These approaches are much more difficult to implement computationally, because *all* atoms must be included. However, in contrast to continuum models, these atomistic models can be used to develop precise models for quantum dot surfaces with defects, lattice relaxation, partial passivation, imperfect interfaces between core quantum dots and capping layers, dots passivated with molecular ligands, and molecularly linked dot arrays. All of these features must be accounted for in the precision modeling tools that are needed to design and implement nanoparticles in specific applications. Hence large-scale implementations of atomistic models for complex nanosystems are essential.

**Fig. 1:**
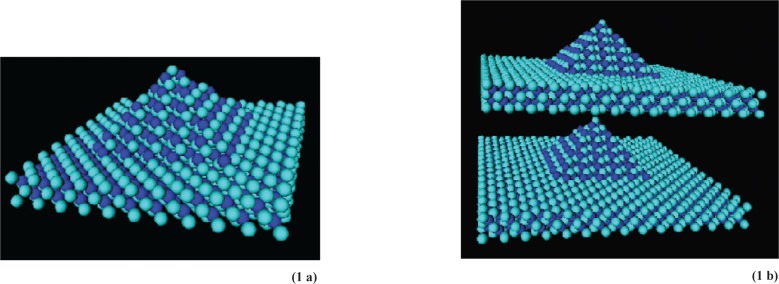
A pyramid structure and a double pyramid structure of InAs quantum dots embedded in GaAs. The surrounding matrix of GaAs is not shown, but would be included in calculations. Coupling between InAs dots is done through the intervening GaAs matrix.

We study the electrical and optical properties of semiconductor nanocrystals and quantum dots such as the pyramidal dot shown in Fig. 1, as well as more complex nanocrystal structures with the nanocrystal coordinated with capping molecules and functionalized with linker molecules, and nanodevice architectures formed by linking together complex dot structures, also shown in Fig. 1.

We use an atomistic approach that allows us to explicitly account for all atoms in the nanoparticles as well as all atoms in any conjugating ligands, linkers and surrounding material. In the most complex structures this entails modeling structures with on the order of a million atoms. Highly parallel computational and visualization platforms are critical for obtaining the computational speeds necessary for systematic, comprehensive study of these structures.

## 2. The Tight-Binding Method and Electronic States of Quantum Dots

Two approaches are now being used to explicitly account for all atoms in a structure. More ab initio theories directly calculate the full electronic states of the system, typically calculating the contribution of each atom to the electronic potential self-consistently within density functional or pseudopotential theory (see for example A. J. Williamson and A. Zunger [6]). This provides the most detailed picture of nanoparticle states but becomes computationally prohibitive for large structures with up to a million atoms. A simpler approach is the tight-binding method.

The tight-binding model [7] is based upon the Linear Combination of Atomic Orbitals (LCAO) method and is both accurate and easy to implement [8, 9]. It finds the electronic states of a quantum dot by assuming that the electronic state can be represented near a given atom by a few atomic orbitals (typically the *s* and *p* orbitals, but in more complete calculations the *s*, *p*, and *d* orbitals) localized to that atom. This assumption makes the calculation significantly simpler than the more ab initio theories but still explicitly accounts for all atoms. The Hamiltonian matrix that describes the coupling between orbitals on neighboring atoms is usually found empirically by adjusting those matrix elements to ensure that bulk band structures are correctly reproduced. Thus there is no need to find an atomic-scale electronic single-particle potential self-consistently. This simplification also greatly speeds up the calculations. Even with these simplifications, the tight-binding theory provides a full atomic-scale theory of complex nanoparticles with monolayer variations in composition capable of accurately describing the structures [9].

Any structure can be studied once the atomic positions are defined. For a nanoparticle that retains the bulk crystal structure, we can start with a large cube of bulk material and throw away all atoms not inside the nanoparticle. Once the position of an atom is determined, its neighbors can be identified. In a tight-binding approach, the orbitals on an atom only couple to its nearest neighbor atoms and possibly to the next nearest neighbors. The short range of the coupling greatly simplifies the computations because the Hamiltonian is sparse. Atomic relaxation away from the bulk positions can also be included, usually with a valence force-field model. Once atomic positions and neighbors are determined for the relaxed lattice, the Hamiltonian coupling matrix can again be determined. Similarly, conjugating and linker molecules can be attached to the nanoparticle. Once the atomic positions in the external molecules are known, the coupling between the nanoparticle and the molecules can be determined. A typical atomic basis would include 10 orbitals. Each atom is described by its outer valence *s* orbital, the 3 outer *p* orbitals, the 5 outer *d* orbitals, and a fictitious excited s* orbital that is included to mimic the effects of higher lying states. When spin is included the basis is doubled. For a system with N atoms, there will be 10 N eigenstates (20 N if spin-orbit coupling is included). For any system except for the very smallest (less than about 1000 atoms), iterative techniques, such as the Arnoldi method [10], must be used to diagonalize the system Hamiltonian and only those eigenstates near the fundamental gap are found. This may mean that 100 to several thousand states are found, but this is still far less than 20 N (where N can be as high as a million or more).

In the tight binding calculations that we typically do, we assume that the atoms in a nanoparticle occupy the sites of a regular zinc-blende or wurtzite lattice. We typically model spherical, hemispherical, tetrahedral, or pyramidal nanoparticles or nanocrystals linked together, e.g., a double quantum dot, quantum dot molecule or arrays of quantum dots.

## 3. The Calculation of Electron and Hole States

### 3.1 Solving the Hamiltonian Equation Sequentially and in Parallel for a Single Nanoparticle

Given that only nearest neighbor coupling is included in our models, the matrix we work with is a large, sparse, banded Hermitian matrix. The “large matrix” we are referring to is the tight-binding Hamiltonian (*H*) whose solutions are the single particle wave functions *ψ_n_* in

$$H\psi_{n}=E_{n}\psi_{n},$$ (1)

where the empirical tight-binding Hamiltonian matrix (*H*) is determined by adjusting the matrix elements to reproduce known band gaps and effective masses of the bulk bandstructures.

Solving this matrix equation for its eigenvalues and eigenvectors (the wave functions) reduces to a matrix diagonalization problem which can be stated as

Az=λz, (2)

where *A* is a large, sparse, Hermitian matrix. The *λ*’s are the energies, and for each *λ*, *z* is an eigenfunction (wave function) for the corresponding energy.

One of the best solvers for this type of matrix diagonalization is ARPACK [10]. In ARPACK, the computational work is proportional to *n* (the size of the matrix is n by *n*, *n* = 10 N or 20 N) times *ncv* (which defines the size of the space to use in finding eigenvectors; we choose *ncv* to be 4 times the number of eigenvalues sought). The only other eigensolver for this class of problem is the Jacobi-Davidson technique [11], but it is *O*(*n*^2^), i.e., the computational work is proportional to *n*^2^ rather than to *n*. There is another consideration, namely, the number of eigenvalues sought. But as long as the number of eigenvalues sought is much less than 20 N, ARPACK is much more efficient. The method employed in ARPACK when *A* is symmetric reduces to a variant of the Lanczos method called Implicitly Restarted Lanczos Method (IRLM) which is a synthesis of Arnoldi/Lanczos with the Implicitly Shifted QR scheme [12]. Implicitly restarted implies iterative, and the matrix is diagonalized iteratively by a series of actions of the matrix *A* on a vector,

$$AV_{k}=>W,$$ (3)

where *W* is part of the workspace in the Arnoldi solver in ARPACK. *V_k_* is the vector representing the single-particle wave function calculated by ARPACK, changing on each iteration *k* (converging to the solution). On the other hand, the action of *A* on *V_k_*, *AV_k_*, is a user supplied matrix-vector multiplication. In the tight-binding code, the matrix-vector multiplication is time-consuming, but the dominant time cost is still the vector reorthogonalization in the Arnoldi solver.

Partial, iterative diagonalization of the sparse Hamiltonian for a nanoparticle benefits greatly from parallelization for the sizes of nanoparticle structures that we deal with. Parallel ARPACK (PARPACK) [13] can be used to explicitly partition and distribute the matrix across nodes of a distributed memory machine (Linux cluster, for example) or processors of a shared memory parallel machine, thereby distributing the workload.

Since PARPACK’s parallelization scheme distributes the Arnoldi vectors *V_k_* across a 1-D processor grid (blocked by rows), we decompose the nanoparticle into slabs such that there are approximately equal numbers of atoms on each processor. Atoms in one slab are coupled to atoms in another slab only via the neighboring atoms at the common interface. Communication between neighboring slabs is minimal while there is no communication between slabs that are further apart. This makes the tight-binding, sparse coupling approach ideal for this parallelization.

The parallel code looks essentially like the sequential code except that the local block of the Arnoldi vector, *V_loc_*, is passed in place of *V*, and *n_loc_*, the dimension of the local block, is passed instead of *n*. There is a user supplied matrix-vector product subroutine to compute the local segment of the matrix-vector product *AV* that is consistent with the partition of *V*. This product requires communication in addition to the communication managed by PARPACK. Memory management is handled by Fortran 90 allocate and deallocate statements so there are parallelization gains coming from the ability to spread the matrices across multiple processors as well as from overall computational time (turnaround) speedup.

**Fig. 2:**
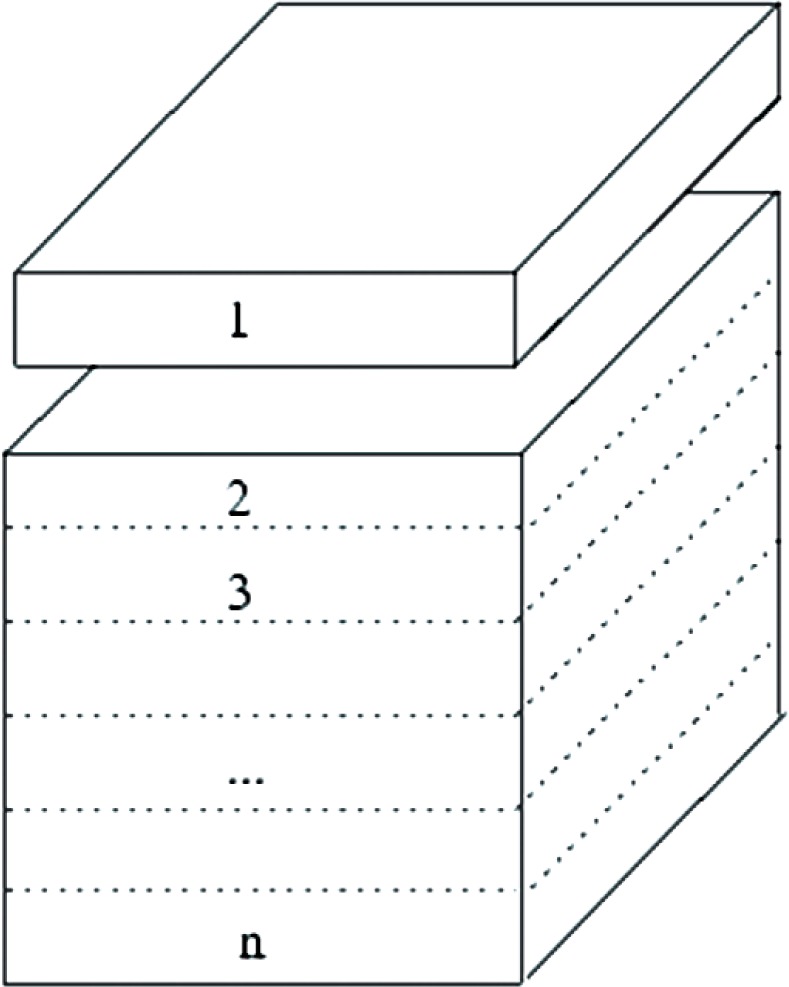
A slab distribution of the computation.

To minimize communication between neigboring slabs, we assume the atoms are ordered in one of the coordinates, which we take to be the *z*-coordinate. We sort and order atoms by *z*, and then catalog *z_min_*,*z_max_* for each processor. We distribute atoms to processors in layers, as depicted in Fig. 2, to more or less evenly distribute the atoms for load balancing.^1^ During the communication phase, each atom on a processor *P* only needs information about atoms which are on *P* plus any atoms which are on neighboring slabs and are the nearest-neighbor distance away from the edge layers on *P*.

**Fig. 3:**
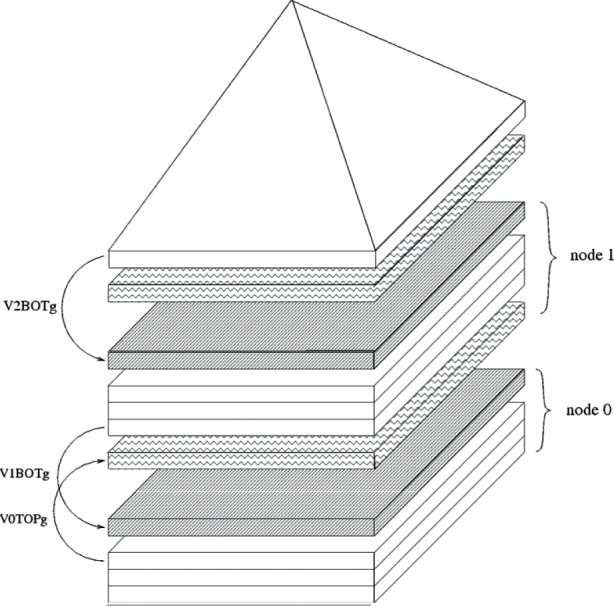
A pyramid structure with ghost layers drawn in. The actual layers and their corresponding ghost layers (shown as shaded layers) are connected by arrows.

For each *P*, we determine the layers in neighboring processors which contain atoms which are nearest neighbors to atoms in *P*. Only the *V*’s for these layers need to be communicated by neighboring processors. So, in Fig. 3, *V*_2_*_BOTg_* is a layer used on processor 1 that is a “ghost” of the bottom layer on processor 2. Similarly *V*_0_*_TOPg_* is a layer used on processor 1 that is a “ghost” of the top layer on processor 0. During the communication phase, processor 1 has only to receive *V*_2_*_BOTg_* from processor 2 and *V*_0_*_TOPg_* from processor 0 for the

$$AV_{loc}=>W_{loc}$$ (4)

product to be computed. Exchanging only these ghost layers and allocating only the actual amount of memory to do this cuts down communication drastically and is the key insight to effective parallelization.

### 3.2 Results

To assess the speedup due to parallelization, we present an example of a diagonalization for an 18 nm diameter spherical HgS nanocrystal with 195393 atoms, each with 5 orbitals. In Table 1 we discuss results for the PARPACK parallelization for the corresponding 976 965 × 976 965 matrix running on the NIST cluster of Pentium, Athlon, and Intel processors running RedHat Linux 2. The run times are for computing twenty different eigenvalue-eigenvector pairs (run times depend on the number of eigenvalue-eigenvector pairs computed).

Notice that simply by sorting in *z* we significantly improved the run time. This speedup is due partly to faster convergence (a smaller number of iterations to convergence), presumably due to better location of the bands in the sparse matrix.

For all of the sorted runs, timings are consistent.^3^ The matrix-vector multiplication is split across processors in an approximately even way. For up to 50 processors, communication is a minor part of the run and CPU time scales. Communication time is 10 to 50 times smaller than the total time. CPU time scales so that the 8 processor time is 5.2 times faster than the 1 processor time. The effects of parallelization are consistent with having a similar number of iterations to convergence for each number of processors. A factor of 25 speedup is achieved on 50 processors for a quantum dot with 195393 atoms, and a matrix size of 976 965 (almost a million). The original sequential job took 2.7 d, the 50 processor job completes in 2.5 h, a significant improvement in turnaround time. Neither communication time nor user-supplied subroutine time is the rate determining step. The bulk of the time and the rate-limiting step is the computation done in PARPACK.

## 4. Building Larger Structures by Stitching

**Fig. 4:**
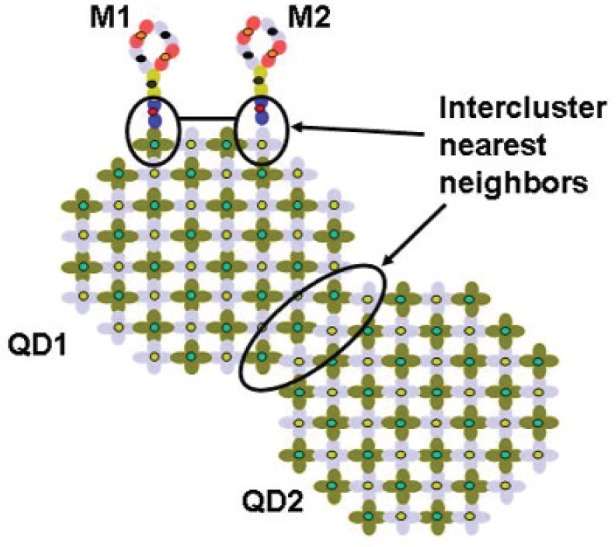
Illustration of intercluster nearest neighbors in a nanosystem with four subsystems: two quantum dots (QD1 and QD2) and two conjugating molecules (Ml and M2).

Often it is easy to define the simple nanosubsystems that make up a complex, heterogeneous nanosystem. However, it may be difficult to explicitly define the entire structure. For example, a single quantum dot is easy to define and implement. An arbitrary sequence of stacked dots is more difficult to implement. However, with the simpler building blocks defined, it is easy to build arbitrary structures with them. A novel feature of our code is the ability to link together heterogenous nanostructures (also referred to here as nanosubsystems). For example, when a nanoparticle nanostructure includes conjugating and linker molecules, these molecules can be assigned separately to different computational nodes to take advantage of the parallelization. If the nanosystem includes multiple smaller nanosubsystems linked together, then each smaller nanosubsystem can be parallelized on a different set of nodes with only minimal communication required between different nodes. In the tight-binding code, the matrix-vector multiplication is important, but the dominant part of the CPU time is the vector reorthogonalization in the Arnoldi solver, which is parallelized efficiently by PARPACK. Hence we are not constrained in how we partition atoms into nanosubsystems (groups in our code, each group having its own cluster of processors), as long as each nanosubsystem partition can be handled as a group. Thus we can choose a partition that conveniently follows the physical partitioning of the structure. Once we can do multiprocessor runs routinely, we have the basic building blocks for making larger structures by “stitching” together disparate subsystems into composite structures, each separate subsystem to be stitched together being a smaller multiprocessor run. The basic idea is to consider each smaller nanosubsystem as its own cluster, using the same input data as before, but at each iteration in the computation, information about atoms in the cluster that are intercluster neighbors (see Fig. 4) has to be distributed to the appropriate processors for the neighboring clusters, thereby “stitching” the calculations on the nanosubsystems in the heterogeneous structure together.

### 4.1 Modifications to the Lattice Generating Code for Stitching

For each small nanosubsystem (group), we build a structure as before, starting with a large cube of bulk material and throwing away all atoms not inside the nanosubsystem. Once the position of an atom is determined, its neighbors in the nanosubsystem can be identified. In the stitching case, each processor needs to know the atom data for each atom in the group (cluster) attached to the processor, as well as all of the atom’s nearest neighbors, whether in its own group or in a neighboring cluster. To reduce communication data between processors, a second pass is done during the lattice generation process to analyze, for each group, the data for all other groups and determine the intra-cluster and intercluster nearest neighbors for each atom. The nearest neighbor table for atoms in each group is augmented by intercluster neighbors (each atom has a global atom id), and data for the intercluster neighbor atoms from other groups is added to the group’s atom data table. This eliminates the need to communicate nearest neighbor data between processors during the iteration phase of the diagonalization.

In the case of a single group, atoms are distributed between processors based on an atom’s *z* position. For a structure with several groups stitched together, each atom is first distributed to a cluster of processors, based on the group that the atom belongs to. Then the atom is distributed to a particular processor in that cluster based on the atom’s *z* position.

### 4.2 Modifications to the Matrix-Vector Multiplier for Stitching

When computing the product in Eq. (4)

$$AV_{loc}=>W_{loc},$$ (5)

at each iteration, diagonal matrix elements present no problem. However, off-diagonal matrix elements require that the *V*’s (eigenvector pieces that change in each iteration) for each atom’s intercluster nearest neighbor atoms (see Fig. 4) must be communicated to the processor who “owns” the atom as in the non-stitching case. Contrary to the non-stitching, one group case where the processors are correlated to the atom *z* position, now we have an irregular grid (where processor atom *z* position is not as well defined) because we stitch arbitrary structures together. With an irregular grid, the communications scheme has to be defined explicitly.

Basically, each processor has to exchange information with all nearest neighbor atoms not on the processor, both from neighbor atoms within its group and outside its group. Initially each processor knows (i.e., can figure out) what processor to send *V*’s to, but not which processors it will be receiving data from or how much data it will be receiving. To solve this problem we use a set of irregular communication routines available from Steve Plimpton[14]. These routines work by first setting up a communication pattern or descriptor and then invoking the communication operation with arbitrary types of data. The end result is that each processor learns how much data it will receive and from whom, so the subsequent communication operations can be performed efficiently. The irregular grid used for stitching heterogeneous nanostructures affects the CPU speedup of our parallel code. In benchmarking stitching code, we find that the parallelization is similar to the non-stitching code illustrated in Table 1 for up to about 40 processors (see Table 2). After that, there is no further speedup as additional processors are included in a run, indicating that for irregular grids communication eventually dominates CPU time and limits the size of a cluster that can be effectively employed in a stitching calculation.

### 4.3 Results

To appreciate the utility of the stitching approach, structures with multiple subsystems must be studied. Two vertically stacked InAs pyramids (quantum dots) on InAs wetting layers embedded in a large GaAs medium (computationally, a large box) have been investigated previously [15, 16] as a single nanostructure. As a test of our stitching code, we have calculated this system as two separate pyramids (each modeled as in Fig. 1) and obtained results for the energy bands and eigenfunctions that are identical to the previous single group calculation. This demonstrates the validity of the stitching code.

As another test, we considered a multilayer nanocrystal structure with a core of CdS, a middle shell of HgS and an outer shell of CdS. Previously these structures have been considered as a single structure [9]. There are six possible combinations of how layers can be ordered for a stitched structure. When considered as a stitched structure, all constructions should give the same results, and they do, again validating our stitching code.

As a more complicated example, we consider three GaAs pyramidal dots (with their wetting layers) stacked on top of each other and embedded in an AlGaAs matrix. Such a structure is a prototypical quantum device with electrons and holes stored in the individual GaAs quantum dots. Charge (either the electrons or the holes) in the outer dots could be brought together in the middle dot to interact and transfer quantum bits (qubits) of information. To design and engineer such devices, it is critical to determine the device tolerance to imperfections in the fabrication. The stitching code greatly facilitates the analysis of such effects.

**Fig. 5:**
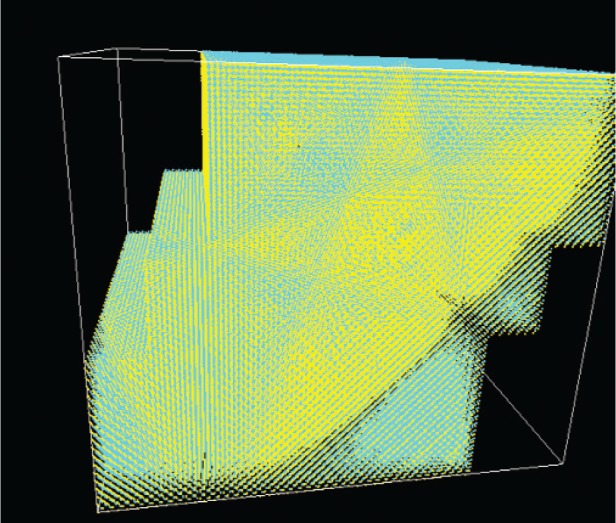
Triple quantum dot structure analyzed by the stitching code. One quantum dot is embedded in each slab (but not visible in the figure). In the perfect structure, the dots would be aligned on top of each other and the corresponding slabs would be aligned. Here the dots are misaligned by the amount corresponding to the slab shift. Different colors represent different anions and cations.

Figure 5 shows the three groups of atoms corresponding to three slabs of AlGaAs, each slab with one embedded GaAs quantum dot and wetting layer. Various alignments of the quantum dots in the structure can be immediately tested simply by shifting the alignment of the slabs, using the same slabs as building blocks for all of the calculations. This greatly reduces the work needed to build different structures, especially if several related structures are to be studied.

**Fig. 6:**
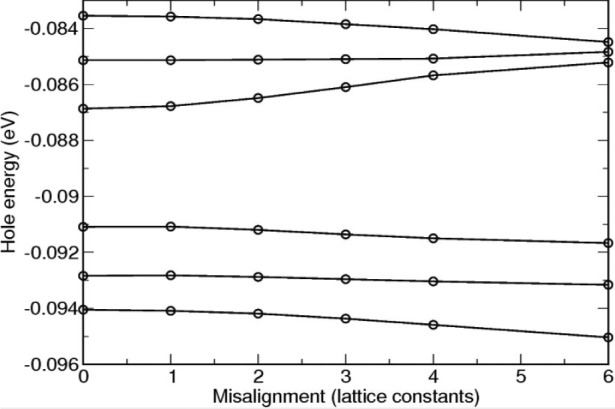
The hole energies of a triple dot structure as a function of the misalignment between adjacent slabs (dots).

The electronic structure of a perfectly aligned structure can be directly determined. For example, the energy levels of the 6 lowest hole states are shown in Fig. 6. To study imperfect structures, we misalign the slabs, redetermine the intercluster nearest neighbor assignments and repeat the calculations. Figure 6 shows how sensitive the levels are to misalignment. The effects become significant for misalignments of 3 or more lattice constants between adjacent dots.

**Fig. 7:**
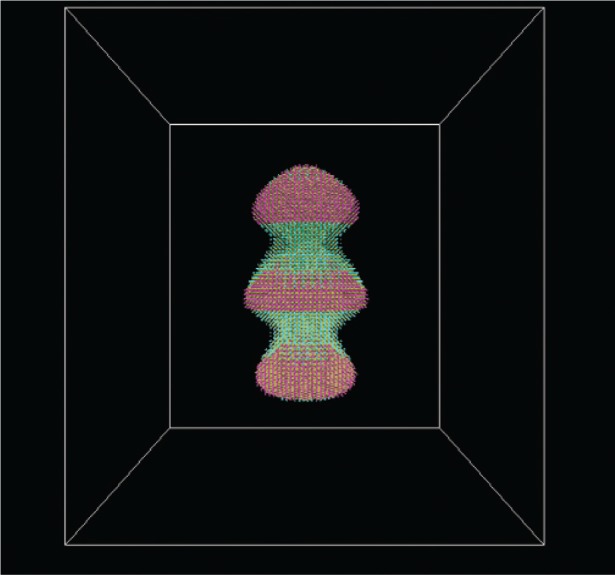
The *s*-orbital charge density of the first electron state in an aligned structure. Different colors denote the charge on anions and cations.

**Fig. 8:**
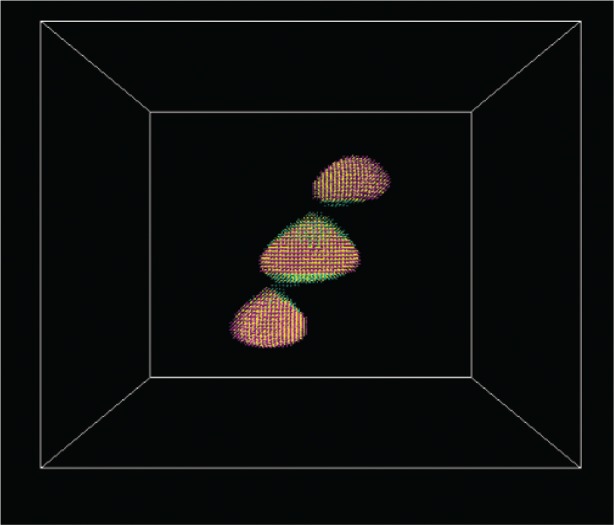
The *s*-orbital charge density of the first electron state in a structure misaligned by 6 lattice constants.

The effects of misalignment are more apparent if the charge densities for the corresponding states are visualized. Figure 7 shows the charge density from the *s* orbitals for the first electron state in the aligned structure, while Fig. 8 shows the corresponding charge density when the adjacent slabs are misaligned by 6 lattice constants, which is nearly the half-width of the dot base and is shown in Fig. 5. In this case the electron state is able to spread between the three dots. This delocalization persists even when the dots are substantially misaligned.

**Fig. 9:**
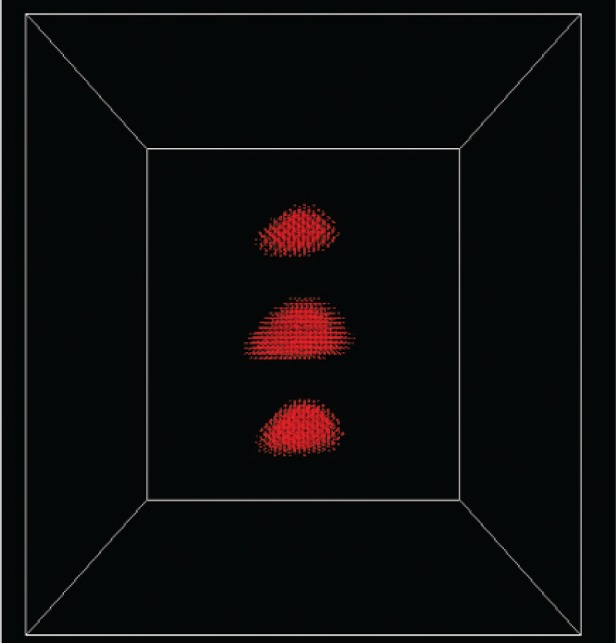
The *p_x_*-orbital charge density of the first hole state in an aligned structure.

**Fig. 10:**
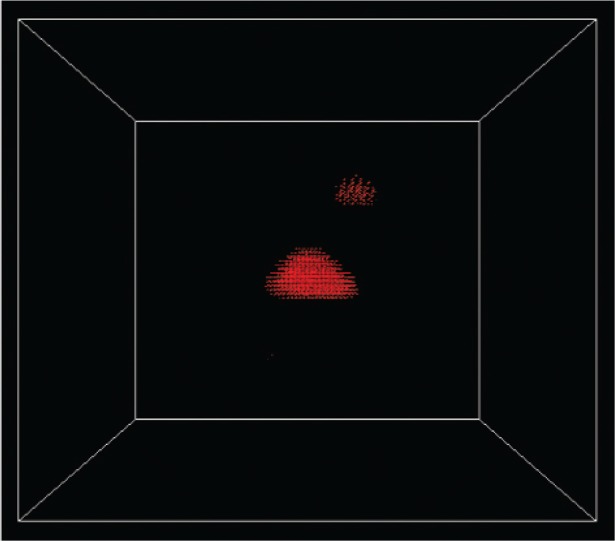
The *p_x_*-orbital charge density of the first hole state in a structure misaligned by 6 lattice constants.

The *p_x_* orbital charge densities for the lowest hole state in the aligned structure and the strongly misaligned structure are shown in Fig. 9 and Fig. 10. In the aligned structure the hole is also delocalized. However, in the misaligned structure the hole becomes strongly localized. Here critical differences in the effects of misalignment on electron and hole states are apparent. Such differences in the consequences of imperfect alignment would be critical in making choices about how to use these structures in quantum devices, as mentioned above. Using these structures as optical devices depends on the electron and hole states having a large overlap. The results show that the overlap is severely impacted by misalignment. Our stitching approach using building blocks to implement and parallelize calculations for large systems makes such studies practical. The calculations we mentioned above did not include *d* orbitals. Including *d* orbitals doubles the number of states per atom. Similarly including spin-orbit coupling doubles the number of states (spin-up and spin-down). In addition the computational demands increase because the spin-orbit coupling requires complex arithmetic. However in a recent study of the electronic structure of GaAs nanocrystals, inclusion of *d* orbitals and spin-orbit coupling proved to be critical to a proper description of the lowest electron states [17]. Hence in these latest calculations, the use of a parallel approach is even more essential. The introduction of *d* orbitals and spin-orbit coupling increases the eigenvector size by a factor of 8 and increases the time for each arithmetic operation (complex arithmetic). Stitching becomes even more critical in this case, because it allows us to take full advantage of multiple processors.

## 5. Visualization

Visual models of laboratory experiments and computational simulations to explore the nanoworld can be critical to comprehension. However, increasing amounts of data are being generated. For example, in the example of the three stacked dots, the region considered has nearly 700 000 atoms. Each atom has 5 orbitals. Thus there are 3.5 million pieces of data to describe one state. Both high performance computing and experiment must be augmented by high performance visualization. At NIST our visual analysis capabilities include both coarse grain capabilities and finer grain capabilities (which are more demanding of CPU and visualization resources) as well as static graphical representations and fully three-dimensional immersive capabilities.

In our quantum dot simulations we visualize the atomic scale structure of the lattice and the charge density of the electrons and holes at both the fine grained and coarser grained levels. Figures in the previous section show one of the finer grained ways we visualize the charge density, i.e., by displaying the contribution of the *s* and *p* orbitals to the charge distribution of an eigenstate of the triple pyramid quantum dot. The orbitals are centered on the atoms in the structure, so these images also represent the atoms in the structure. This visualization is important because the presence of significant orbital charge density between the dots indicates that tunneling is probable between the structures, i.e., the visualization shows the tunneling created by coupling dots in the structure.

**Fig. 11:**
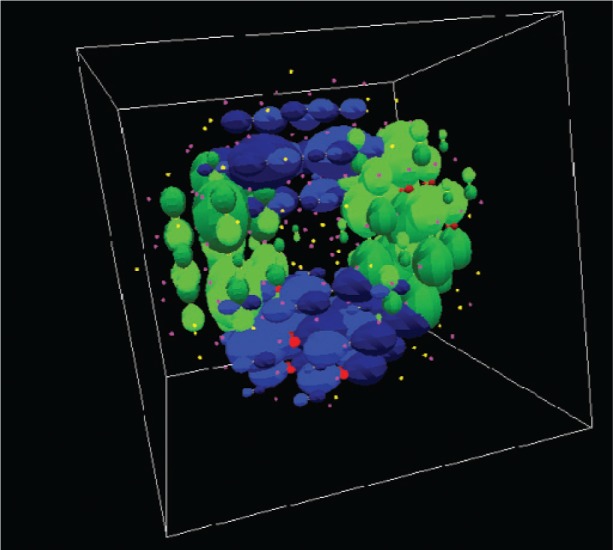
Charge density of the lowest hole state in a CdS nanocrystal.

Finer detail than the detail visible in Figs. 7–10 can be represented in a visualization. Figure 11 shows the charge density of the lowest hole state in a CdS nanocrystal. In this case, much greater detail is apparent. The contributions from *p_z_* orbitals (green) and *p_x_* orbitals (blue) are shown. The contributions of *p_y_* and *s* orbitals are not visible in this example. The orbitals are centered on the corresponding atom. The shape, size, and color represent the orbital type and the magnitude of its contribution. The different colors of the orbital lobes (for example, lighter and darker blue for *p_x_*) indicate the phase of the orbital. In this way, complete information about electron and hole states can be obtained. For example, state symmetries can be discerned immediately from these visualizations. Such symmetries are more difficult to discern otherwise.

**Fig. 12:**
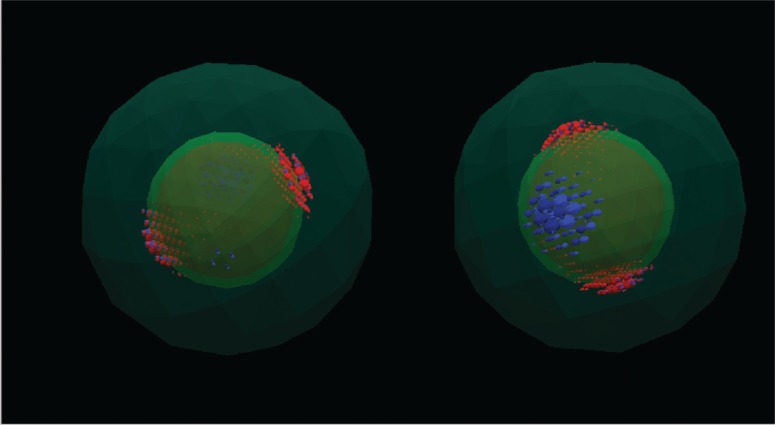
Two different views of atomic state density of an electronic state trapped in the well region of a nanoheterostructured nanocrystal.

Even for these examples, the amount of data to be visualized can be prohibitive. Coarser grained visualizations can avoid that problem. Figure 12 is an example of contours and transparent surfaces which shows charge densities in a coarser grain way. The figure shows the atomic scale charge density of an electronic state trapped in the well region of a CdS/HgS/CdScore/well/clad nanoheterostructured nanocrystal.

**Fig. 13:**
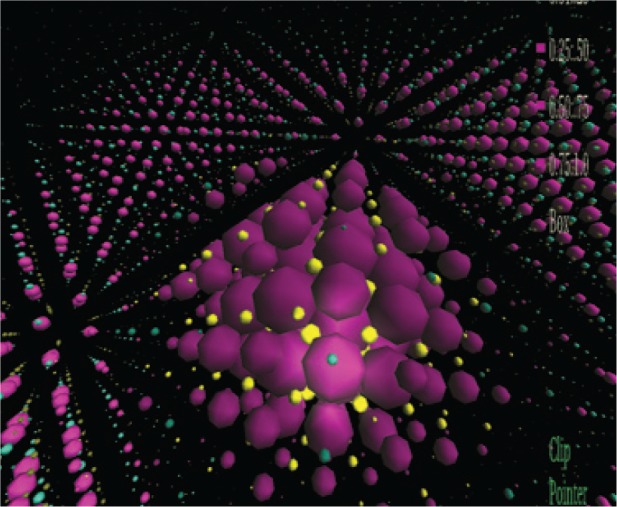
Snapshot from an immersive visualization of a quantum dot. The spheres represent *s* orbitals, which also are representative of the atoms in the structure.

We can do much more with the output of our nanostructure calculations. Our visual analysis capabilities include an immersive environment that allows scientists to interact with their data by navigating through a three-dimensional virtual landscape of the data rather than by simply viewing pictures of the data. Our nanostructure calculations output detailed charge distributions which are transferred to the NIST immersive environment where they can be studied interactively. One can move through space, going inside the structure and moving around inside the structure. In this way one can visualize the structure looking in from the outside, or looking out from the inside. One can visualize both the nanostructure (see, for example, Fig. 13), and the atomic scale variation of calculated nanostructure properties from any orientation and position in space. This is not possible with any static graphical representation. For an example of an interaction with a nanostructure in the immersive environment (which can be saved as a quicktime movie), see [18].

Our representations are tremendously helpful. They encapsulate the physics and allow one to easily see features that might be missed by just perusing the voluminous output from a supercomputer size calculation. Such insights are very helpful and greatly speedup the extraction of useful understanding and insights as we explore the properties of new and unfamiliar systems.

## 6. Summary

In this article we discuss the use of high performance computing and visualization for the simulations of the nanoscale systems that would be used in emerging nanotechnologies, biosensors and quantum devices. Paradoxically, the properties of individual nanostructures often depend on their atomic scale structure while the complex device structures used in these nanotechnologies integrate multiple nanosystems and contain a million or more atoms. One must have a multiscale computational approach that allows one to routinely study systems with a million atoms or more, including the atomic scale detail. We use the tight-binding approach to include atomic-scale detail. We use code parallelization to make million atom calculations feasible. We have implemented a stitching approach to the parallelization to allow us to implement and study efficiently complex nanosystems built from heterogeneous building blocks. To mine the voluminous amounts of data that are generated, we used a variety of fine-grained and coarse-grained approaches that span the range from static representations to immersive visualization. The later allows us to move interactively to regions of high interest in complex structures to rapidly identify and isolate key features. However, immersive visualization still comes at the cost of tremendous hardware demands to run the immersions. Thus simpler representations still play a critical role in gaining insights into the physics and operations of these nanotechnologies and quantum devices.

## Figures and Tables

**Table 1:** Computation and communication times in seconds to find 20 eigenvalues of a 976 975 × 976 975 matrix as a function of the number of processors

Number of processors	user-supplied matrix-vector subroutine	communication time (s)	CPU time (s)	Number of iterations
1 (unsorted)	13525		778420	1016
1 (sorted)	9710		228470	863
2	4616	1957	111720	788
4	2464	2065	64065	890
8	1405	1075	44122	877
10	1353	1194	46314	1154
16	592	1365	17244	780
32	323	605	14568	925
40	275	394	11267	761
50	225	117	9239	800

**Table 2:** Computation times for two groups stitched together as a function of the number of processors

Number of processors	CPU time (s)
4	155402
8	81910
16	59905
32	34826
40	20869
50	25697
80	36600

## Footnotes

1 Because we put all atoms with the same *z* on the same processor, the number of atoms on different processors may be different. In practice the difference between the maximum and minimum number of atoms in a layer is about 10 %.

2 Certain commercial equipment, instruments, or materials are identified in this paper to foster understanding. Such identification does not imply recommendation or endorsement by the National Institute of Standards and Technology, nor does it imply that the materials or equipment identified are necessarily the best available for the purpose.

3 There is an anomaly at 10 processors which is due to the number of iterations to convergence. This is because convergence depends on the choice of initial vector and ARPACK’s choice of an initial vector is different for different numbers of processors. We find a 10 % to 30 % deviation in our runs coming from this factor.
